# Can Helicopters Solve the Transport Dilemma for Patients With Symptoms of Large-Vessel Occlusion Stroke in Intermediate Density Areas? A Simulation Model Based on Real Life Data

**DOI:** 10.3389/fneur.2022.861259

**Published:** 2022-04-25

**Authors:** Anne Behrndtz, Richard Beare, Svitlana Iievlieva, Grethe Andersen, Jeppe Mainz, Martin Gude, Henry Ma, Velandai Srikanth, Claus Z. Simonsen, Thanh Phan

**Affiliations:** ^1^Department of Neurology, Aarhus University Hospital, Aarhus, Denmark; ^2^Department of Medicine, School of Clinical Sciences at Monash Health, Stroke and Ageing Research, Monash University, Melbourne, VIC, Australia; ^3^Department of Clinical Medicine, Prehospital Department, Aarhus, Denmark

**Keywords:** Large-Vessel Occlusion, prehospital/EMS, helicopter, rurban area, model, transport-air, mothership, drip & ship

## Abstract

**Background:**

This modeling study aimed to determine if helicopters may optimize the transportation of patients with *symptoms* of large vessel stroke in “intermediate density” areas, such as Denmark, by bringing them directly to the comprehensive stroke center.

**Methods:**

We estimated the time for the treatment of patients requiring endovascular therapy or intravenous thrombolysis under four configurations: “drip and ship” with and without helicopter and “bypass” with and without helicopter. Time delays, stroke numbers per municipality, and helicopter dispatches for four helicopter bases from 2019 were obtained from the Danish Stroke and Helicopter Registries. Discrete event simulation (DES) was used to estimate the capacity of the helicopter fleet to meet patient transport requests, given the number of stroke codes per municipality.

**Results:**

The median onset-to-needle time at the comprehensive stroke center (CSC) for the bypass model with the helicopter was 115 min [interquartile range (IQR): 108, 124]; the median onset-to-groin time was 157 min (IQR: 150, 166). The median onset-to-needle time at the primary stroke center (PSC) by ground transport was 112 min (IQR: 101, 125) and the median onset-to-groin time when primary transport to the PSC was prioritized was 234 min (IQR: 209, 261).

A linear correlation between travel time by ground and the number of patients transported by helicopter (rho = 0.69, *p* < 0.001) indicated that helicopters are being used to transport more remote patients. DES demonstrated that an increase in helicopter capture zone by 20 min increased the number of rejected patients by only 5%.

**Conclusions:**

Our model calculations suggest that using helicopters to transport patients with stroke directly to the CSC in intermediate density areas markedly reduce onset-to-groin time without affecting time to thrombolysis. In this setting, helicopter capacity is not challenged by increasing the capture zone.

## Introduction

Reducing delay to the reperfusion therapy has been pivotal in stroke management since the publication of time-dependent outcomes for both intravenous thrombolysis and thrombectomy (1, 2). Techniques for a faster diagnosis of acute ischemic stroke (AIS) have been investigated in rural and rural-urban areas and include telemedicine (3), prehospital stroke scores (4), and point-of-care tests (5). Furthermore, in urban areas, mobile stroke units (6) are operating. The helicopter transport of patients with stroke has been reserved for rural and mountainous areas and the use of air transport for shorter distances has been avoided due to the delay caused by organizing air retrieval (7). Many European countries, such as Denmark, have a population density at the rural-urban-continuum (8) with some confluent townships (9). Intermediate density has been accepted as the terminology for this population density, this is widespread throughout the European continent. The definition of intermediate density areas is that less than 50% of the population lives in regions considered as rural (minimum 300 inhabitants/km^2^) and less than 50% of the population lives in regions considered as urban (maximum 1,500 inhabitants/km^2^) (10). The few observational studies in intermediate density areas investigating the use of helicopters have shown mixed effects (11–13).

Since endovascular therapy (EVT) became the standard of care for patients with large vessel occlusions (LVOs) (11–15), prehospital triage with clinical scales to detect LVO has been developed (16–18). Computed transport modeling (19, 20) and ongoing randomized trials (21, 22) have questioned if the “bypass model” with direct transport to a comprehensive stroke center (CSC) outperforms the “drip and ship” model with initial transport to the nearest primary stroke center (PSC) and subsequent referral to a CSC in the case of LVO. The potential risk associated with the bypass model is increased time to intravenous thrombolysis (IVT) for the patients with non-LVOs (23). The previous mapping methods have not investigated this in intermediate density areas or explored the consequences of helicopters with *fast dispatch* (19, 20, 24). This study aimed to analyze time delays for the different transport strategies for patients with possible LVO located in the intermediate density areas where helicopters with fast dispatch are used. This approach could be of use in other intermediate density area settings.

## Methods

### Geography

Denmark has a total area of 43,000 km^2^ and consists of the Jutland peninsula and 78 inhabited islands. The 2020 population was 5.8 million. Stroke service is provided by seven PSCs and three CSCs (25). One of the PSCs is functionally a CSC during working hours. Ground emergency medical ambulances are equally distributed across the country, and the median response time is 8 min for patients with stroke (26).

### Helicopters

Three helicopters were established permanently on three bases in 2014 and a fourth base was added in 2018 to reduce the delay for emergency patients to specialized hospital services (SHSs). In Denmark, level 1 trauma care, PCI, and EVT are located at the same four Danish centers providing SHS (27).

**Figure 1:**
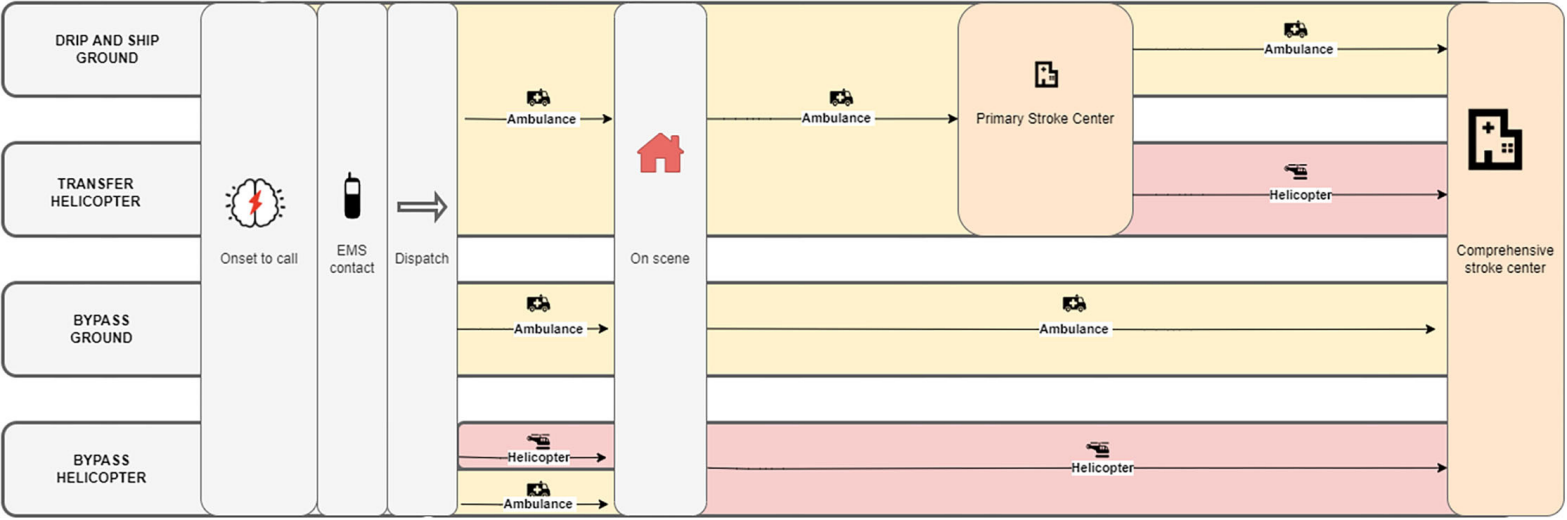
Comparing ground transport to helicopter transport to the nearest comprehensive stroke center (CSC). In the helicopter model, an ambulance is dispatched at the same time as the helicopter, and the ambulance personnel has prepared the patient for helicopter transport (minimum 28 min).

The helicopters are operational 90% of the time despite challenging weather conditions, owing to improved new technologies, including night vision goggles and night vision imaging systems (28). The base is staffed 24/7, and a reserve team is available if the active team reaches operational limits (29). Patients with acute medical illnesses call the regional Emergency Medical Service (EMS), which consists of both EMS health professionals and an Emergency Medical Dispatch Center. Since 2014, the helicopter has been dispatched whenever a patient presents with the symptoms of severe stroke (30, 31), and the staff deems that the patient would benefit from helicopter transport to a CSC (32). The emergency staff activate simultaneous ambulance and helicopter dispatch while the patient or bystander is on the call (Figure 1). A geographical dispatch protocol is not defined in Denmark. If ambulance personnel arrive first, they prepare the patient while waiting for the helicopter. Rendezvous is preferred if the ambulance is waiting for the helicopter to arrive. The helicopter is dispatched only for transport to CSCs and for urgent transfer from a PSC to a CSC. Helicopter dispatch time, i.e., time to airborne is 5 min. Ambulance dispatch time is 1 min.

## Analysis

A schematic diagram (Figure 1) outlines the comparisons of ground and helicopter transport. Four scenarios are described: (1) using only ground transport and the “drip-and-ship approach” (going to a PSC first), (2) using a helicopter for transfer taking the “drip-and-ship approach,” (3) using ground transport in the “bypass approach” (going directly to a CSC), and (4) using a helicopter in the “bypass approach.”

The time from dispatch to the scene for EMS ground transport was simulated in this model. Valid random addresses dispersed over the whole country were sampled from the Danish Address Registry, ten from each of the 98 municipalities. Addresses, helicopter bases, and hospitals were geocoded using the tmaptools (R-package interface to OpenStreetMap Nominatim). Ground transport time from the addresses to the nearest PSC and CSC and between the PSC and the CSC were calculated using googleway R-package interface to Google Maps Application Programming Interface (API). The optimistic transport option was specified in the model to simulate ambulance travel time. We sampled the times in morning peak hours, at lunch, at afternoon peak hours, and at night.

Helicopter trip times were calculated using the distance between start and end locations and assuming an average helicopter speed of 240 km/h using sf package (R package), this includes take-off and landing. This approach was chosen because registered timestamps in the helicopter is a field with imprecise and missing registrations, due to obvious errors in the manual data entry procedure (33). We assumed that the ambulance was dispatched, had arrived at the scene, and had used the median time at the scene before the helicopter arrived. We further assumed that in this model the helicopter was always able to land close to the patient without rendezvous. This assumption was based on a previous study showing that more than 92% of all landings are within 500 m from the patient (34). Our median on scene delay for the helicopter is based on an ongoing study of patients with acute stroke and ST-elevation myocardial infarction (STEMI) (35). Transfer time from the PSC to CSC was estimated as the sum of helicopter dispatch time, time from helicopter base to the PSC, and from the PSC to the CSC, as shown in Figure 1.

Information on additional delays came from annual reports and published data from the Danish Stroke Registry and the Danish helicopter EMS (HEMS) database (Table 1) (29, 36). The median time from onset to call was 30 min and the time on scene for EMS ground personnel was 20 min. When using the helicopter, an additional 11 min were used to load the patient. The latter is based on median times from the helicopter registry. It is assumed that the patient is prepared by the ground EMS for air transport. Air transport from the scene cannot be initiated less than 28 min from the emergency call. In this case, only ground transport is preferred (this corresponds to the large catchment area in discrete event simulation [DES]). At both CSC and PSC, door-to-needle time was 27 min. Door-to-groin-puncture when using the bypass approach was 68 min, whereas door-to-groin-puncture was 41 min when using the drip-and-ship approach. Door-in-door-out at the PSC was 60 min.

**Table 1:** Onset to treatment for patients in the catchment area of a primary stroke center (modeled from 671 centroids).

	**Drip-and-ship**	**Bypass with ground**	**Bypass with helicopter**
Onset to IVT (minutes)	112 (101,125)	140 (118,162)	115 (108,124)
Onset to EVT (minutes)	234 (209,261)	181 (159,203)	157 (150,166)

*Median with interquartile range (IQR). Time delay for EVT is significantly shorter when bypassing with helicopter, whereas delay is not affected for IVT treatment. EVT, Endovascular therapy; IVT, Intravenous thrombolysis*.

### Existing Dispatch Patterns

The geographical dispatch pattern that has developed in the absence of specific geographic dispatch rules was analyzed using data from the annual reports correlated with our data at the municipality level (Appendix Tables 2, 3). We used shapefiles from Eurostat (37). Air and ground transport times from all municipalities to the nearest SHS were calculated using the methods described above. The geographical dispatch pattern was analyzed focusing on transport times as a function of the number of dispatched helicopters per municipality. Islands without bridges were excluded from this analysis, as air transport is the only transport option in these islands.

### Discrete Event Simulation

Discrete event simulation is a method to model a sequence of discrete events in time and was performed to evaluate if the helicopter can handle the transportation load. We simulated the maximum helicopter trips per year based on the maximum possible LVOs, LVO mimics, and other acute conditions, such as heart attacks and trauma (Appendix). SHSs and CSCs are located at the same centers. If the waiting time exceeds 15 min, the patient was placed in a “queue.” The patient was “rejected” to seek an alternate transport (e.g., ground transport) if the waiting time exceeded 1 h. The input was a median 180 min base-to-base flight time, the calculated flight times from the model above.

In the DES, three helicopter catchment zones were defined. The largest zone is when ground transport is covering an area of less than 28 min to the nearest SHS. This time is based on the median time to ambulance arrival plus the median time on scene. The middle and small catchment zone is when ground transport is covering 38 to 48 min from the scene to SHS. Additionally, we tested a scenario with two helicopters per base using the DES model. For the simulation, we used the simmer R package and we made a thousand simulations for each model (38). In the DES, the assumptions were that the medical staff would operate around the clock, that helicopters operated 90% of the time, and that the number of flights for patients diagnosed with other conditions has not changed from 2019. In 2019, one helicopter was flying at an average of 2.8 times per day. An average trip is estimated to be 165 min from leaving the base until return to base. Refueling is not included in this analysis.

### Statistical Analysis

Non-parametric analysis (Wilcoxon signed-rank test) was used to analyze the non-normally distributed data.

## Results

**Figure 2:**
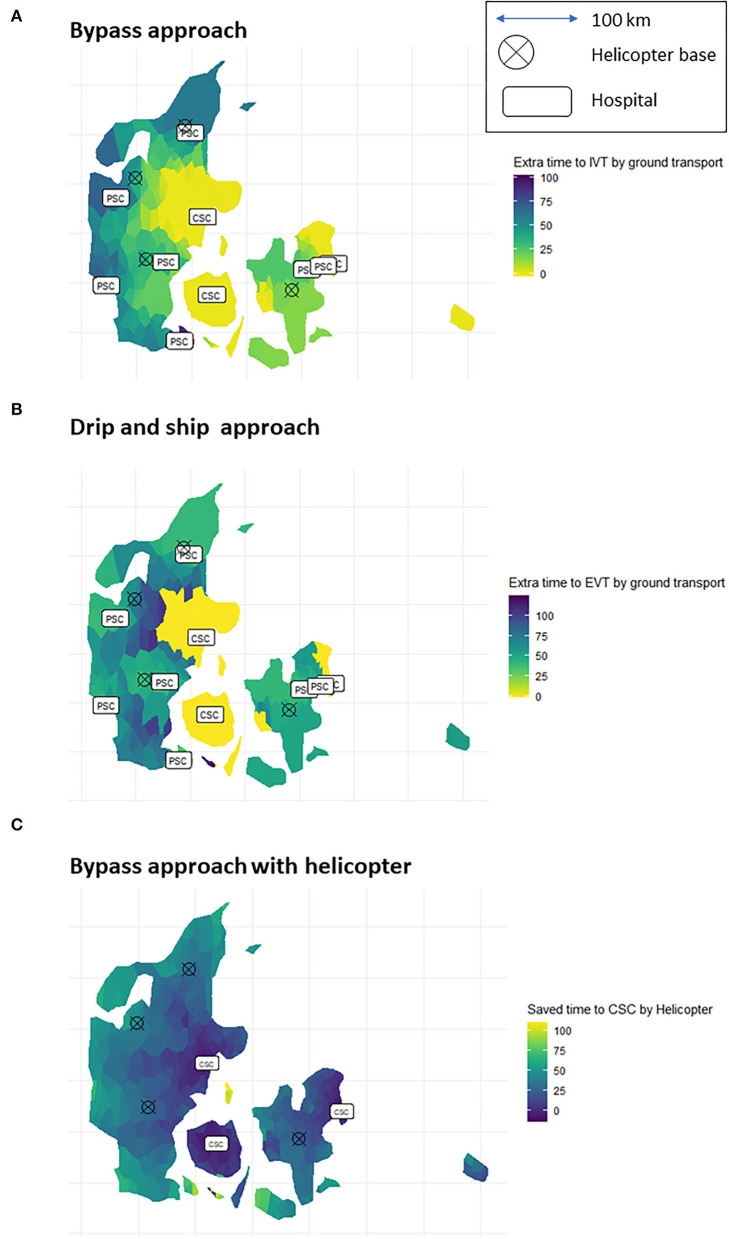
Additional time to intravenous thrombolysis (IVT) with bypass approach **(A)** and additional time to IVT with the drip and ship approach **(B)**. Time saved to revascularisation therapy at a CSC when using the helicopter **(C)**. The brighter colors the shorter delays.

The median ground transport time from the scene to the nearest PSC was 26 min (IQR: 16–39), and the map shows an equal geographical distribution of ground transport times to the nearest PSC (Appendix Figure 1). Median ground transport time from the scene to the nearest CSC was 50 min (IQR: 33–77) for patients in the catchment areas of a PSC. No significant difference (*p* < 0.05) in PSC transport time was observed according to the time of the day. The overall median transport time to the CSC in the afternoon and at night was 8 min (*p* < 0.05) longer than morning and midday because only three CSC centers are open during off-hours (Appendix Tables 4, 5). When prioritizing the “drip and ship” method, the time to the EVT of patients located on the border between a PSC and a CSC was delayed by more than 100 min, and when prioritizing the “bypass” method, the time to the IVT of patients situated geographically farthest away from the CSCs was delayed by more than 75 min (Figures 2A,B).

Time from onset to IVT at a CSC with the helicopter (bypass approach) is 115 min (IQR 108–124) compared with 112 min (IQR 101–125) at the ground to the nearest PSC (drip and ship approach). For patients with LVO, the time from onset to groin puncture with the “bypass model” approach was 156 min (IQR: 149–165) compared with 234 min (IQR: 209–262) when first transported by ground to the nearest PSC (drip and ship). These delays were all statistically different from each other (*p* < 0.05). The increased delay was due to both prolonged transport time and door-in-door-out delay at the PSC (Table 1). Patients situated further away from the CSCs saved more time than those situated close to the CSCs (Figure 2C). When stratifying for PSC, the time delay for patients with non-LVO was prolonged by 4–14 min depending on PSC. At one PSC, the catchment area time was reduced (by 6 min) when using the helicopter. The time saved for EVT treatment varies by 51–120 min depending on the PSC catchment area (Appendix Tables 6, 7).

When helicopters are used to transfer patients between the PSC and the CSC, the time saved using the helicopter varies from 4 min in the Capital area (considered urban) to 44 min in the southern region. More time is saved when the longer the distances are from the PSC to the CSC, and the most time-saving transfers are seen from the four PSCs that have the longest ground transport time and/or the shortest direct straight-line distance (Appendix Table 8).

**Figure 3:**
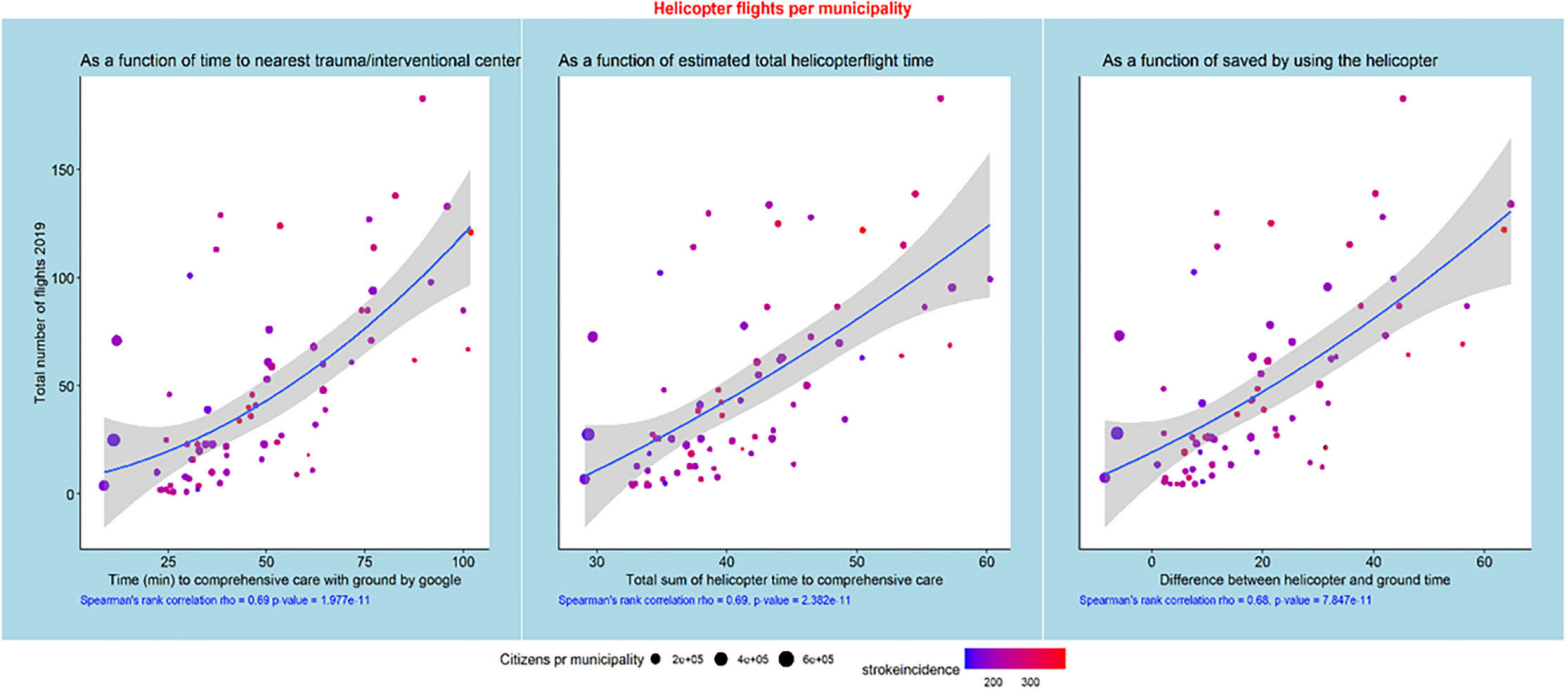
Helicopter flights per municipality as functions of ground transport time, total estimated helicopter time, and the difference between ground transport and helicopter transport times. The municipalities with the highest numbers of citizens are close to the CSCs, but there is a trend toward a high incidence of strokes far away from the CSCs.

The helicopter dispatch pattern is shown as the number of flights to each municipality as a function of time to SHS estimated by Google ground transport times, as a function of the total sum of helicopter time to SHS, and as a function of the difference between total helicopter flight time and ground transport time (Figure 3). A significant linear relationship is observed (rho = 0.69, *p* < 0.001, rho = 0.69, *p* < 0.001, and rho = 0.68, *p* < 0.001) in all three cases. Moreover, the figure also shows that larger cities are closer to the CSCs, whereas smaller cities are further away. The cities further away tend to have a higher stroke incidence, illustrated by red color (as shown in Appendix Figures 2, 3).

**Figure 4:**
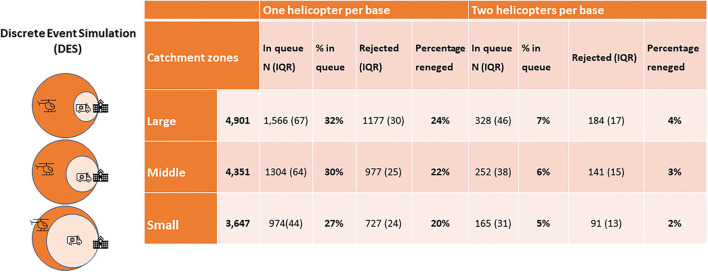
Results of the discrete events simulation of 1000simulations. Showing the number of patients rejected and placed in a queue (>15min) awaiting transport to specialized hospital services (SHS). Large, 28 minutes to SHS with ground transport; Middle, 38 minutes to SHS with ground transport; Small, 48 minutes to SHS with ground transport. SHS, specialized hospital services; IQR, interquartile range.

Discrete event simulation was used to estimate helicopter capacity in a large, middle, and small helicopter catchment zone. When the helicopter catchment area increases, the ground transport catchment area decreases. Figure 4 shows that the number of rejected patients increases from 20 to 24% when increasing the helicopter zone from small (48 min to SHS by ground) to large (28 min to SHS by ground). When having a small catchment zone 27% of the patients wait more than 25 minutes, and this number increases to 32% when having a large catchment zone. If two helicopters were available per base then the percentage of rejected patients would drop to 5–7% depending on the size of the catchment zone and the number of patients in the queue will drop to 2–4%.

## Discussion

The key findings of this study are that the helicopter transport directly to CSC significantly shortened the time to EVT (from 234 to 156 min) without prolonging the time to IVT markedly (112 vs. 115 min) for patients who can only be treated with thrombolysis. This is mainly due to a short dispatch time for helicopters, strategically placed helicopter bases, and a reduced flight transport time compared to ground transport.

A prospective before-and-after study comparing the drip-and-ship approach to the bypass approach made *only* in the Central Denmark Region (30, 39) had similar findings: time delay to groin puncture for the patient with LVO was significantly reduced in the bypass approach (185 vs. 234 min in the drip-and-ship model). In this study, the time delay for IVT was even improved (112 min. for the bypass model; 119 min for the drip-and-ship model) probably due to the already established helicopter transport for patients with stroke when conducting this study. Another observational study from the *same region* reported a 17–54 min time saving on PCI and trauma patients for helicopter transport (13). Two observational studies from Eastern Denmark have shown no effect of using a helicopter on PCI and EVT (11, 40), which corresponds to our findings. The reason might be the degree of urbanization of this region. The strength of using a model compared to using observational studies is the ability to compare different transport strategies for the same geographically located patient.

The American Heart Association (AHA) guidelines suggest that the PSC should be bypassed if transport time to the CSC is less than 15 min longer than transport time to the PSC (41). According to this model, all Danish patients with putative LVOs should go directly to a CSC when the helicopter can be dispatched (Appendix Table 7). In this setting, a prehospital stroke severity scale with a higher sensitivity but a lower specificity may be considered because bypassing will not prolong the time to IVT. The consequence of this will be more non-LVO admissions to the CSC. The transfers of patients with LVO are difficult to avoid due to the relatively low sensitivity of stroke severity scales. When studying helicopter transfers between the PSC and the CSC in this model, we find that time is saved for transport from six of the seven PSCs.

In Denmark, EMS is allowed to ignore traffic lights and speed limits and use all available lanes provided they are careful (42). Real travel times might therefore be faster than Google API times. This is a limitation to this paper and a project addressing this issue is ongoing in the Central Danish Region. The relationship between ambulance travel times and estimates using Google APIs needs to be understood before using this approach in other locations. If the ambulances are not allowed to avoid traffic laws, the ground transport time by ambulance could very well correspond to Google API as documented in, for example, Australia (24).

This model could also be used in more dense areas (megapoles) during rush hours but it requires a well-organized prehospital structure (43). The usage of HEMS performance inside the metropole for traffic victims has lately been described in Buenos Aires and does require coordination between police and EMS to ensure public safety (44). Air transport could also be relevant in cases where the patient is situated outside the megapole in a less dense area where landing facilities are good. This must be further explored.

No settled operational guideline exists for the dispatch of the helicopter according to the geographical location of the patient. Therefore, we analyzed the helicopter dispatch pattern in Denmark in 2019 and observed a correlation between the actual use of the helicopter and our calculated times saved by using the helicopter. Despite the lack of geographical dispatch algorithms, the EMS dispatchers seem to dispatch on the right geographical basis.

The potential case load that the existing fleet of helicopters might service is an important question that we investigate *via* DES. This analysis pertains to only putative LVOs and other hyperacute conditions. We performed DES to assess the capacity of the system to handle the large caseloads of patients with putative LVO. DES is used in industry and businesses to evaluate bottlenecks in system and capacity but it is rarely used in stroke (45). Our findings suggest that the four helicopter bases in Denmark can handle this case load very well but a considerable number of patients (30%) have to wait in the queue for more than 15 min. Furthermore, having 2 helicopters per base would reduce the number of patients having to wait in a queue. The model can therefore be used to explore the details of helicopter fleet deployment and the impact of changing aircraft numbers. When extrapolating these results to other rural area settings, extending geography does not seem to overwhelm capacity but queuing appears to be an issue with few flights per day. A solution could be more helicopters per base.

A formal cost-effectiveness analysis was beyond the scope of this study. However, the effectiveness of helicopter transport can be interpreted in the context of the conditional probability of an outcome. Investigators have used conditional probability models to calculate the optimal transport strategy based on time-dependent outcomes (1, 19). When using the newest model (20), the probability of an excellent outcome changes from 0.29 to 0.31 when shifting from a drip-and-ship ground model to a bypass ground model, rising to 0.32 when the helicopter was employed in the bypass model. An absolute increase in a good outcome of 3% results in a “number needed to fly” of 33 to achieve a *good outcome*. This corresponds well with the previous outcome depending measures on thrombectomy alone (2). “Numbers needed to fly” for a *better outcome* at 3 months for all patients with AIS is 20 using these measures. If implemented elsewhere, the helicopter transport of patients with stroke may cause additional expenses. Additional costs for one patient getting a *better outcome* will be approximately 152 thousand euros. We suggest that the benefits of shifting for a better outcome will be carried out in a cost-effectiveness analysis. Trials exploring the use of mobile stroke units in intermediate density areas have not been published, but using them in rural areas has been proposed (46).

## Conclusion

This modeling study suggests the timely benefit of using helicopters with a fast dispatch in an intermediate density area where overcoming long distances and mountains are not a challenge in the prehospital setting. When using a helicopter, bypassing the PSCs seems beneficial for patients with the symptoms of LVO stroke. More than an hour was saved for patients with thrombectomy patients, while no additional delay to IVT for patients with non-LVO was seen. In this setting, the helicopter capacity is not challenged by expanding its use to more extensive geography, but it could be optimized by having two helicopters per base.

## Data Availability Statement

The original contributions presented in the study are included in the article/Supplementary Material, further inquiries can be directed to the corresponding author.

## Author Contributions

AB gathered all open source information. Prehospital information was confirmed by MG. The relevant analysis was made by AB with help from TP, RB, and SI. AB wrote the first draft with help from CS. All authors reviewed and edited the manuscript and approved the final version of the manuscript.

## Conflict of Interest

The authors declare that the research was conducted in the absence of any commercial or financial relationships that could be construed as a potential conflict of interest.

## Publisher's Note

All claims expressed in this article are solely those of the authors and do not necessarily represent those of their affiliated organizations, or those of the publisher, the editors and the reviewers. Any product that may be evaluated in this article, or claim that may be made by its manufacturer, is not guaranteed or endorsed by the publisher.
