# Supplementary material for: Can Helicopters Solve the Transport Dilemma for Patients With Symptoms of Large-Vessel Occlusion Stroke in Intermediate Density Areas? A Simulation Model Based on Real Life Data
Source: Front Neurol. 2022 Apr 25;13:861259. doi: 10.3389/fneur.2022.861259 (PMC9082641; doi:10.3389/fneur.2022.861259)
Supplement: Supplementary file 1 [file Data_Sheet_1.pdf]

Appendix

Can Helicopters solve the Transport Dilemma for Patients with Symptoms of Large-Vessel Occlusion Stroke in Intermediate density Areas?

A simulation model based on real life data

Methods

Table1.Times, numbers and sources used in the model:

Median times (min)		source		
onset-EMS call (patient delay)	30min	Jeppe Mainz 2015-17	only for those who are getting reperfusion treatment	
Ambulance response time	8min (1min disopatch+7min drive)	Jeppe Mainz 2015-17	only for those who are getting reperfusion treatment	
On scene time	20min	Jeppe Mainz 2015-17	only for those who are getting reperfusion treatment	
Ground transport time	google trip times	Sampled for Wednesday the 16.09.2020 (The country was "open" as usual, nationwide covid restrictions for 2. wave sat in the 20.09.2020)		
Door-to-needle-time median Denmark	27min	DAP annual report 2018 p 34		
DIDO	60min	TRIAGE-STROKE interim		
Door-to-groin mothership	68min	Goliath(Århus trial)		
Door-to-groin drip and ship	41min	Goliath(Århus trial)(68-27=41)		
EMS call to airborne	4,5min	https://www.akutlaegehelikopter.dk/om-os/besatning/		
Helicopter transport time	4km/min	https://www.akutlaegehelikopter.dk/om-os/helikopter/		
Time at scene helicopter	11min	Preliminary result from the helicopter database Gowry		

Table 2 and 3. Summary of annual report findings

**Helicopter missions in 2019**	n
Patients airlifted	2005
Patients attended by doctor on scene (not lifted)	918
Missions aborted	1307
**Total**	4230

**Strokes 2019**	
Strokes	10586
EVT-treated	670
IVT-treated	2224

Hems technologies:

HEMS fly with a cruising speed of 240km/h, they are using night vision googles, GPS Pins corridors for landing, cloud break procedure and IFR-route network.

Results:

Table 4. Time to nearest CSC depending on time at day:

Characteristic	AfternoonPeakTime, N = 980[1]	LunchTime, N = 950[1]	MorningPeakTime, N = 950[1]	NightTime, N = 980[1]
Time to nearest CSC	44 (25, 72)	36 (24, 60)	37 (24, 62)	44 (26, 71)
Unknown	0	0	1	1

[1] Statistics presented: Median (IQR)

Table 5. Time to nearest stroke center depending on time at day:

Characteristic	AftenoonPeakTime, N = 950[1]	LunchTime, N = 950[1]	MorningPeakTime, N = 951[1]	NightTime, N = 950[1]
Time to nearest strokecenter	26 (16, 39)	26 (16, 39)	26 (16, 40)	27 (16, 39)
Unknown	0	1	1	0

[1] Statistics presented: Median (IQR)

Figure 1. Distribution of times to IVT by ground transport at nearest stroke center:

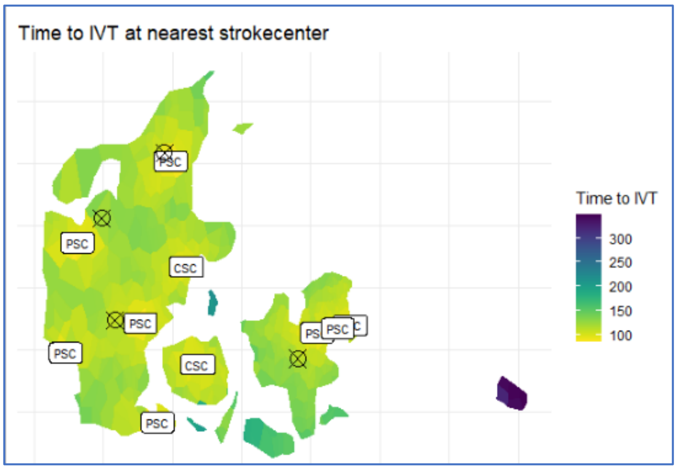

Table 6. Time delay to treatments when stratifying for PSC catchment area:

Characteristic	AalborgHospital, N = 80[1]	AarhusHospital, N = 91[1]	BlegdamsvejHospital, N = 96[1]	EsbjergHospital, N = 48[1]	GlostrupHospital, N = 186[1]	HolstebroHospital, N = 63[1]	OdenseHospital, N = 92[1]	RoskildeHospital, N = 172[1]	SoenderborgHospital, N = 36[1]	VejleHospital, N = 86[1]
Time to IVT (DS)	117 (108, 124)	118 (108, 132)	102 (97, 111)	119 (111, 131)	100 (96, 110)	116 (102, 124)	113 (105, 123)	122 (108, 133)	116 (105, 132)	110 (106, 116)
Time to EVT (DS)	259 (250, 266)	159 (149, 173)	143 (138, 152)	275 (266, 286)	197 (194, 207)	268 (254, 276)	154 (146, 164)	231 (217, 241)	280 (269, 297)	230 (226, 236)
Time to IVT(MS)	163 (148, 179)	119 (108, 132)	102 (97, 111)	177 (167, 196)	110 (106, 114)	162 (151, 179)	113 (105, 123)	141 (128, 152)	175 (159, 191)	131 (123, 141)
Time to EVT(MS)	204 (189, 220)	160 (149, 173)	143 (138, 152)	218 (208, 237)	151 (147, 155)	203 (192, 220)	154 (146, 164)	182 (169, 193)	216 (200, 232)	172 (164, 182)
Time to IVT (heli)	129 (123, 134)	119 (108, 132)	102 (97, 111)	132 (128, 133)	106 (105, 108)	127 (122, 129)	113 (105, 123)	116 (112, 120)	119 (116, 120)	116 (113, 118)
Time to EVT (heli)	170 (164, 175)	160 (149, 173)	143 (138, 152)	173 (169, 174)	147 (146, 149)	168 (163, 170)	154 (146, 164)	157 (153, 161)	160 (157, 161)	157 (154, 159)

[1] Statistics presented: Median (IQR)

Table 7. Time saved/lost with drip and ship versus helicopter bypass model and ground bypass model versus helicopter bypass model.

Characteristic	AalborgHospital, N = 80[1]	AarhusHospital, N = 91[1]	BlegdamsvejHospital, N = 96[1]	EsbjergHospital, N = 48[1]	GlostrupHospital, N = 186[1]	HolstebroHospital, N = 63[1]	OdenseHospital, N = 92[1]	RoskildeHospital, N = 172[1]	SoenderborgHospital, N = 36[1]	VejleHospital, N = 86[1]
Timedifference to IVT (DS-helicopter MS)	-14 (-19, -5)	0 (0, 0)	0 (0, 0)	-10 (-20, -1)	-5 (-8, 3)	-13 (-25, -1)	0 (0, 0)	6 (-5, 15)	-4 (-11, 9)	-4 (-9, -1)
Timedifference to EVT (DS minus helicopter MS)	87 (81, 96)	0 (0, 0)	0 (0, 0)	105 (95, 113)	51 (48, 59)	97 (86, 110)	0 (0, 0)	73 (63, 82)	120 (113, 132)	75 (70, 78)
Time saved using the helicopter in mothership appoach	34 (25, 44)	0 (0, 0)	0 (0, 0)	46 (36, 64)	4 (1, 7)	36 (29, 47)	0 (0, 0)	24 (16, 32)	53 (38, 65)	14 (10, 23)

[1] Statistics presented: Median (IQR)

Table 8 Transfer times for helicopter versus ground transport:

PSCtransfer_from	time_base_hospital	heli_transfer_min	heli_all_transfer	ground_transfer_min	difference
AalborgHospital	2.4	23.9	31.3	67.5	-36.2
EsbjergHospital	13.4	30.3	48.7	81.8	-33.1
GlostrupHospital	11.3	2.9	19.2	23.3	-4.0
HolstebroHospital	7.7	25.0	37.7	77.8	-40.1
RoskildeHospital	7.3	7.7	20.0	34.3	-14.3
SoenderborgHospital	24.9	15.9	45.8	90.2	-44.4
VejleHospital	5.8	16.0	26.9	46.0	-19.1

Figure 2. Helicopter trips per year per municipality in 2019. Red dots are helicopter bases blue dots are comprehensive stroke centers.

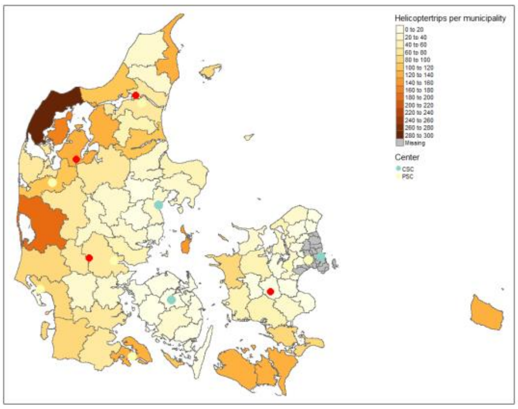

Figure 3. Calculated LVOs per municipality per year in 2019. Red dots are helicopter bases and blue dots are comprehensive stroke centers.

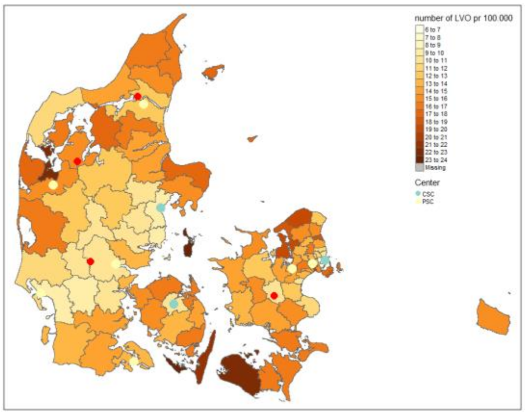

Discussion

Figure 5. We used Venema et. al. calculations for probability of excellent outcome. As outcome for non LVO patients was with NIHSS less than 10 with Holodinsky model:

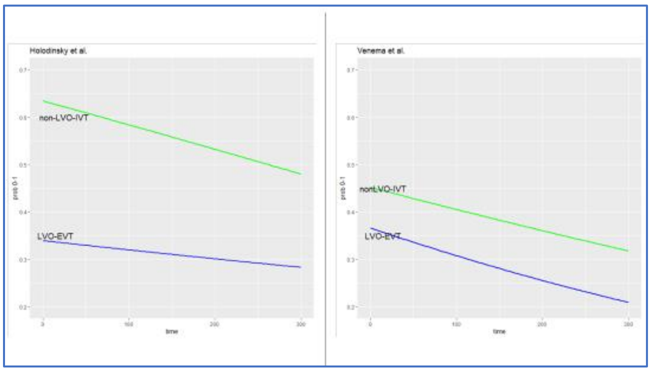

We used Venema et. al. calculations for outcomes:

Given AIS and all gets treated and calculated with median times for both mothership and drip and ship model:

LVO and EVT=0.89*(plogis(1.35 +(- 0.0026*Time to groin+log(0.13/0.87)))

LVO and IVT reperfusion=0.11*(plogis(1.35 +(- 0.0026*Time to IVT+log(0.13/0.87)))

Non LVO and IVT=plogis(0.56 +(- 0.0019*Time to IVT)+log(0.32/0.68))

All=0.68*LVO+0.32*non LVO

<u>Helicopter Costs (ref 32 main article)</u>

All costs (fuel, base, staff, and maintenance) 24,3mio Euro year. Cost per helicopter treated patent were 24,3mio Euro/2923patients=8313Euro/patient - if all costs are equally distributed at <u>all airlifted</u> patients attended by the helicopter.

670 thrombectomies out of a population on 5827463 (statbank.dk, 2019 4Q) makes the incidence of LVO 11/100.000 people gets EVT treatment (ref DAP and statbank.dk). We need to fly 4 patients for each patient who gets thrombectomy (reference TRIAGE data 2020). Hence 44/100.000 people per year needs to be air lifted -in catchment areas for the helicopter.

In case of all putative LVOs getting helicopter, we should fly 670*4=2680patients but the actual number of flights were 400(20%) with neurological disease. Hence, we are only flying 15% of our putative strokes probably because ground transport is used for the rest. With the LVO screening

tool we use[33] and Danish EVT rate this would cost 3.7MEuro/100.000inhabitants/year in the catchment areas for the helicopter.

We assume the airlifted patients and the patients treated at scene cost the same.

Cost of airlifted patients in 2019: 2008patients*8313Euro=16,7MEUR

Currently approximately 15% of patients with symptoms of a LVO stroke are airlifted with helicopter in Denmark. Expenses for one helicopter trip is 8313 Euro and compared with one ambulance transport at 668 EUR. The additional costs for the helicopter setting in the catchment areas of the helicopter is 3.7million EUR/100.000 inhabitants/year.

Saver et. al: 39 out of 1000 patients be less disabled at 3 months for each 15 minutes delay. Reduced delay for thrombectomy in this trial is 77minutes. This result in 200/1000 patients getting a better outcome after thrombectomy. We need to fly 200/100*4= 20 patients for 1 getting a better outcome

Additional costs for using the helicopter is (8313-668)*20= 152900 EUR

Table 9. Estimated helicopter costs:

	Percentage of the transferred patients	The actual cost of the patients per diagnose in 2019
**Heart disease**	42%	7,0 MEUR
**Trauma**	23%	3,8 MEUR
**Neurological disease**	20%	3,3 MEUR
**Medical disease**	10%	1,7 MEUR
**Gastrointestinal disease**	4%	0,7 MEUR
**Other**	1%	0,2 MEUR
**Total**	100%	16,7MEUR

Ambulance costs:

Costs ambulances per year 282467533EUR/422760trips=668EUR per ambulance ride.
Ref:
https://www.regioner.dk/services/nyheder/2018/oktober/brug-for-mere-konkurrence-paa-ambulancedrift-18-forslag-til-regionerne

https://www.sundhed.dk/content/cms/56/101656_aarsrapport_praehospitaldatabasen_2019_offentliggjort.pdf

(Price Danish crones: 2000000000/422760=4730kr pr trip)
